# Concentration, Propagation and Dilution of Toxic Gases in Underground Excavations under Different Ventilation Modes

**DOI:** 10.3390/ijerph19127092

**Published:** 2022-06-09

**Authors:** Javier Menéndez, Noe Merlé, Jesús Manuel Fernández-Oro, Mónica Galdo, Laura Álvarez de Prado, Jorge Loredo, Antonio Bernardo-Sánchez

**Affiliations:** 1 Mining and Civil Department, SADIM Engineering, 33005 Oviedo, Spain; 2 Mining and Civil Department, Duro Felguera, Parque Científico Tecnológico, 33203 Gijón, Spain; noe.merle@durofelguera.com; 3 Fluid Mechanics Department, University of Oviedo, 33271 Gijón, Spain; jesusfo@uniovi.es (J.M.F.-O.); galdomonica@uniovi.es (M.G.); 4 Department of Mining Technology, Topography and Structures, University of León, 24071 León, Spain; laura.alvarez@unileon.es (L.Á.d.P.); abers@unileon.es (A.B.-S.); 5 Mining Exploitation Department, University of Oviedo, 33004 Oviedo, Spain; jloredo@uniovi.es

**Keywords:** toxic gases, underground excavations, tunneling, blasting, numerical modeling, field measurements

## Abstract

The drill-and-blast method is widely used for the excavation of hard rock tunnels. Toxic gases such as carbon monoxide and nitrogen oxides are released immediately after blasting by the detonation of explosives. To provide a safe working environment, the concentration of noxious gases must be reduced below the threshold limit value according to health and safety regulations. In this paper, one-dimensional mathematical models and three-dimensional CFD numerical simulations were conducted to analyze the concentration, propagation and dilution of the blasting fumes under different operating conditions. Forced, exhaust and mixed ventilation modes were compared to determine the safe re-entry times after blasting in a 200 m-long tunnel excavated using the top-heading-and-benching method. Based on the numerical simulations, carbon monoxide was the most critical gas, as it required a longer ventilation time to reduce its concentration below the threshold limit value. The safe re-entry time reached 480 s under the typical forced ventilation mode, but was reduced to 155 s when a mixed ventilation system was used after blasting, reducing the operating costs. The reduction of the re-entry time represents a significant improvement in the excavation cycle. In addition, the results obtained show that 1D models can be used to preliminary analyze the migration of toxic gases. However, to reliably determine the safe re-entry times, 3D numerical models should be developed. Finally, to verify the accuracy of the CFD results, field measurements were carried out in a railway tunnel using gas sensors. In general, good agreements were obtained between the 3D numerical simulations and the measured values.

## 1. Introduction

Toxic gases are produced when the drill-and-blast method is used to excavate underground infrastructures. The noxious gases released immediately after blasting are mainly carbon monoxide (CO) and nitrogen oxides (NOx) [1]. These gases must be diluted and removed to provide a safe working environment for the workers. Therefore, a suitable ventilation system is required to reduce the concentration of the gases below the threshold limit value (TLV) according to health and safety regulations [2]. The safe re-entry time mainly depends on the ventilation system employed, the ventilation airflow and the distance from the working face to the tunnel outlet [3]. Forced ventilation is normally used in tunneling construction [4]. However, there are other ventilation systems that can also be used to reduce the re-entry times. Researchers have previously analyzed the migration of toxic gases generated to determine the safe re-entry time after blasting. Computational fluid dynamics (CFD) modeling is typically used by researchers to predict the concentration–time curves of the noxious gases after blasting in underground excavations. Huang et al. [3] analyzed the evolution of the CO concentration after blasting in a copper mine. They considered a forced ventilation system in a 15 m^2^ mining tunnel under different working conditions and concluded that the distance between the mouth of the ventilation duct and the working face, as well as the ventilation airflow, influences the dispersion coefficient of the toxic gases in the tunnel. Bahrami et al. [5] developed an advection–dispersion transport model to evaluate the safe re-entry time after blasting using a gas-monitoring system in the return of the ventilation system in a limestone mine. They concluded that the installation of a monitoring gas system can be used to reliably determine the safe re-entry time of the workers after blasting. Torno et al. [6] developed conventional and CFD numerical models to explore the migration of the blasting fumes in a coal mining heading. They validated the numerical results with field measurements to study the safe re-entry time of the miners to the heading face. Feng et al. [7] numerically analyzed the dynamic diffusion of CO after blasting in a high-altitude tunnel using forced ventilation. They concluded that the time required to reduce the CO mass fraction increases when the underground infrastructure is located in high-altitude environments. Other studies have also been developed in underground coal mines to study blasting fume dilution using auxiliary ventilation systems [8]. Pu et al. [9] constructed a 3D CFD numerical model to simulate the diffusion process of toxic gases after blasting in a 47 m^2^ railway tunnel using forced ventilation. They used different ventilation duct layout modes and different tunnel construction methods, such as full-face excavation and the top-heading-and-benching approach. Huang et al. [10] carried out a study on the environmental impact of drilling and blasting in tunneling construction in Norway, analyzing the effects of the tunnel length and size. They concluded that the drilling-and-blasting process causes a hazardous environment to the health of construction workers.

To improve the mining cycle, mathematical and empirical models have also been used by researchers to investigate the propagation of blasting fumes and the re-entry times after blasting operations [11,12]. Agson Gani et al. [13] developed a study to determine the re-entry time after blasting in an underground gold mine. They analyzed the dispersion of the CO, the diffusion coefficient and the mine ventilation system at different locations. Sirait et al. [14] studied the effective advection–diffusion coefficient to evaluate the time required to remove the toxic gases after blasting using gas detectors in an underground mine. Gillies et al. [15] developed a tool based on a gas-monitoring system and proposed mathematical models to evaluate safe re-entry times. Harris and Mainiero proposed the application of negative pressure to a borehole to remove the CO near the blast [16]. Other researchers have also explored the CO and NO migration in blasting operations, analyzing the adverse health effects for workers [17,18]. The concentrations of toxic gases and dust in the working area depend on the forced ventilation layout and the tunnel construction method [19,20]. The air velocity was also analyzed by Li et al. in large underground caverns [21]. They concluded that air velocities greater than 0.15 m s^−1^ are required to reduce the CO concentration below the TLV. Zhang et al. [22] conducted a study on the distribution of CO and dust in mining headings and concluded that fan selection had a great importance for removing the toxic gases generated by the detonation of explosives. Other researchers have also studied the migration characteristics of toxic gases during the excavation of underground infrastructures in high-altitude areas [23,24] and concluded that the time required to dilute the toxic gas concentration decreases at lower altitudes. Chang et al. [25] investigated the forced ventilation system during the construction of a roadway tunnel by applying CFD modeling. Different configurations of duct position, air velocity in the duct and distance from the mouth of the air duct to the tunnel face were examined to determine the CO concentration. CFD species transport models are also used to analyze the thermal environment and the control of dust during tunneling excavation under different ventilation systems. Comparative studies were carried out by Xin et al. [26] to investigate the cooling performance during the excavation of underground headings under different ventilation systems. They considered a 40 m-long tunnel with two air ducts of 0.8 m in diameter and different lengths in a forced-exhaust ventilation system. A CFD analysis was developed by Bubbico et al. [27] to study the toxic species in roadway tunnels by considering a standard k-ε turbulence model. The formation and control of dust were examined under different operating conditions. They argued that the distance between the ventilation duct and the working face had a great importance on the dust control [28,29]. The re-entry time after blasting has also been studied in a room-and-pillar mine by considering the concentration of NOx released from an ANFO blast [30]. Currently, gas-monitoring systems in underground mines are used successfully to predict the re-entry times after blasting operations [31]. CFD numerical simulations are also carried out for evaluating the risk level in road tunnels under fire scenarios while considering natural ventilation [32,33].

In this paper, the propagation and dilution of the toxic gases released after blasting operations during the excavation of a railway tunnel, using the top-heading-and-benching method, were investigated. The concentration of the toxic gases generated after blasting must be reduced below the TLV according to safety and health regulations. In order to optimize the excavation cycle time, different ventilation systems were analyzed to reduce the safe re-entry time after blasting. Forced, exhaust and mixed ventilation modes were employed in a 200 m-long tunnel equipped with an 1800 mm-diameter ventilation duct. To improve the efficiency of the ventilation system, a 1000 mm-diameter short-forced duct was also considered in the mixed system close to the working face, together with the main exhaust duct. Analytical and three-dimensional CFD numerical models were used to predict the concentration–time curves of the toxic gases after blasting at different cross sections. The species transport model was employed to consider a mixture of gases composed by CO, NO, NO_2_ and air. Finally, to verify the accuracy of the CFD simulations, field measurements of the CO mass fraction were carried out with a stationary gas sensor in a railway tunnel.

## 2. Methodology

### 2.1. Problem Statement

**Figure 1:**
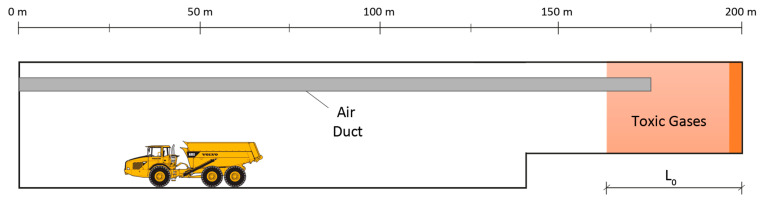
Longitudinal profile of the tunnel, with the ventilation duct and the toxic gases located in the tunnel face after blasting.

Ventilation systems are required in underground excavations to supply fresh air into the working face, dilute the combustion gases emitted by diesel engines and provide a suitable and safe working environment for the workers. According to the Spanish regulations, the amount of air required depends on the number of workers (0.04 m^3^ s^−1^ worker^−1^) and the power of the combustion engines (0.04 m^3^ s^−1^ HP^−1^) [8]. In addition, toxic gases, mainly CO, NO and NO_2_, are produced after blasting operations in tunneling excavation. These gases also have to be diluted using the ventilation system. After blasting operations, the construction workers must remain outside the tunnel until the blasting fumes are removed using the ventilation system. The time required to reduce the toxic gas concentration below the TLV according to safety regulations is known as the re-entry time. The re-entry time depends on the ventilation system, the distance from the tunnel face to the tunnel outlet and the ventilation airflow [5]. A forced ventilation system is typically used in tunneling construction, but there are other systems that can also be used to reduce the re-entry times and improve the operating cycle. A 200 m-long railway tunnel with a cross-sectional area of 68 m^2^, excavated using the top-heading-and-benching method, has been considered. The longitudinal profile of the tunnel after blasting is shown in Figure 1. Immediately after blasting, the toxic gases were concentrated at the tunnel face (z = 200 m), occupying a volume with length *L*_0_, which can be empirically estimated depending on the amount of explosives [3].

**Figure 2:**
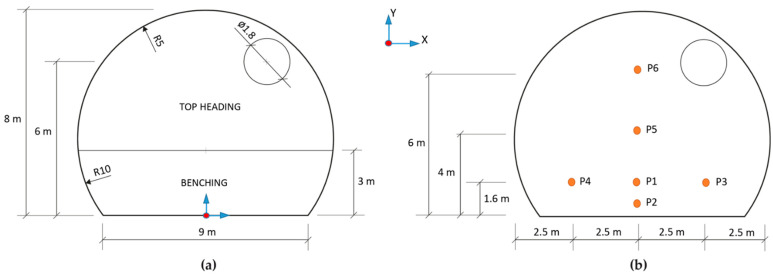
Cross-sectional area of the railway tunnel excavated by the top-heading-and-benching method: (**a**) the section’s geometry and ventilation duct and (**b**) the locations of the observation points.

The geometry of the tunnel’s cross section is shown in Figure 2. The top heading was 5 m high and 10 m wide, with a cross-sectional area of 39 m^2^. The total excavation height was 8 m and the cross section of the full face was 68 m^2^. An 1800 mm-diameter ventilation duct was considered at 6 m high in the right haunch. In addition, Figure 2b shows the locations of six observation points in the cross section to analyze the distribution of the CO, NO and NO_2_ mass fraction with time. Note that points P1, P3 and P4 were located at the height of the human breathing zone (Y = 1.6 m) and point P2 was located at a height of 0.8 m.

**Figure 3:**
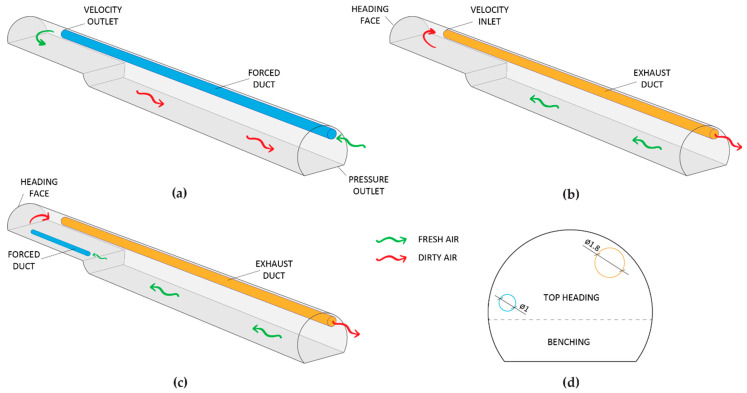
Schematic diagram of the ventilation systems: (**a**) forced system; (**b**) exhaust system; (**c**) mixed system; and (**d**) cross-sectional area of the mixed ventilation system.

Three ventilation systems with an axial fan located at the tunnel portal were considered. A schematic diagram of the ventilation systems is depicted in Figure 3. Figure 3a shows the forced ventilation system. The fresh air entered through the forced duct and reached the working face. After blasting, the toxic gases and dust were diluted and returned through the tunnel itself towards the outside at a lower velocity. The exhaust ventilation mode is presented in Figure 3b. The fresh air entered through the tunnel and reached the working face. The air backflow with the blasting fumes returned through the exhaust duct towards the tunnel outlet (z = 0 m). Note that the exhaust system requires the installation of a wire-reinforced duct and a dust collector. Finally, a mixed ventilation system is shown in Figure 3c. A short-forced air duct with a 1000 mm-diameter and an additional axial fan located at a distance of 60 m from the working face were used to reduce the re-entry time after blasting. The locations of the air ducts are indicated in the tunnel cross section in Figure 3d. The fresh air entered through the tunnel and the additional axial fan blew the air up to the tunnel face. The blasting fumes and dust returned through the exhaust air duct to the tunnel outlet. Airflow rates of 30 and 40 m^3^ s^−1^ were considered in forced, exhaust and mixed ventilation modes, while 15 m^3^ s^−1^ was supplied by the short-forced duct in the mixed ventilation system. The distance between the mouth of the forced and exhaust ducts and the tunnel face was 25 m. However, the distance from the short-forced duct to the working face in the mixed system was reduced to 20 m.

### 2.2. Initial Concentration of Toxic Gases

CO, NO and NO_2_ were mainly produced after blasting operations in the top heading of the tunnel. The throwing length of the hazardous gases immediately after tunnel blasting operations depends on the amount of explosives and can be obtained by applying Equation (1), where *L*_0_ is the throwing length (m) of the toxic gases and *G* is the quantity of explosives (kg) [3]. The initial concentration of toxic gases was calculated using Equation (2) [3]. CO is the initial gas concentration (%), *G* is the amount of explosives, *b* is the volume of gas produced per kilogram of explosive (m^3^ kg^−1^), *M_GAS_* is the molar mass of the gas (g mol^−1^), *A* is the cross-sectional area of the top heading (39.27 m^2^) and *M_AIR_* is the molar mass of air (28.96 g mol^−1^). Emulsion explosives with a volume of gas per kilogram of explosive of 0.014, 0.00125 and 0.00065 m^3^ kg^−1^ for CO, NO and NO_2_, respectively, have been considered [34,35]. Finally, the distance from the air duct to the tunnel face (*L_e_*) was estimated empirically by applying Equation (3) [3].

(1) $$L_{0}=15+\frac{G\;}{5}$$

(2) $$C_{0}=\frac{G\;b\;M_{GAS}}{L_{0}\;A\;M_{AIR}}$$

(3) $$L_{e}=4\;\sqrt{A}$$

### 2.3. Threshold Limit Values (TLV)

Table 1 shows the exposure limits for CO, NO and NO_2_ according to the Spanish mining safety regulations (ASM-2) [8]. The time-weighted average (TWA) and the short-term exposure limit (STEL) are indicated. The TWA is the time-weighted average concentration of a toxic substance over a normal 8 h work day and 40 h work week. The STEL is the acceptable exposure limit to a toxic substance over a period of 15 min. In addition, Table 2 presents the exposure limits for CO according to international safety and health regulations, such as NOHSC (National Occupational Health & Safety Commission, Australia), NIOSH (National Institute for Occupational Safety and Health, Washington, DC, USA) and OSHA (Occupational Safety & Health Administration, Washington, DC, USA). 

### 2.4. Analytical Model

A one-dimensional model was developed by Taylor to analyze the dispersion of matter in a turbulent flow through a straight long pipe [36,37]. This model, known as the one-dimensional advection–diffusion transport model, allows the unsteady evolution of the concentration to be calculated at any point in the space using intrinsic coordinates that are moving at the advection velocity. In the case of a geometry with a single characteristic cross section, the analytical solution of the model is given by Equation (4), which has been previously used in the literature to investigate the propagation of blasting fumes in underground excavations [38,39]:

(4) $$C\;\left(z,t\right)=\frac{V}{2A\sqrt{\pi Dt}}\exp\left(\frac{-{\left(z-\bar{u}t\right)}^{2}}{4Dt}\right)$$

where *C (z, t)* is the concentration of gas at position *z* and time *t*, *V* is the volume of gas at the original state (*z* = 0, *t* = 0), *t* is the time from the release of the contaminant (s), *A* is the cross-sectional area of the tunnel (m^2^), *z* is the distance from the source (m), *ū* is the uniform flow velocity (m s^−1^) and D is the effective diffusion coefficient (m^2^ s^−1^). Note that Equation (4) predicts the toxic blasting gas concentration at any distance from the source and time based on the critical parameter *D*. Precisely, Taylor [37] proposed an additional equation to estimate an equivalent diffusion appropriate for a 1D domain according to the following expression:

(5) $$D=5.05\;D_{H}\;\bar{u}\sqrt{\frac{f}{8}}$$

where *D_H_* is the hydraulic diameter (m) of the tunnel and *f* is the friction factor (-). The friction factor can be obtained according to any of the typical empirical correlations employed in the literature [40]. In particular, as shown in Equation (6), the Von-Kárman equation for fully rough flow has been considered as the most convenient for turbulent flow in underground excavations as a function of the wall roughness ε (m):

(6) $$\frac{1}{\sqrt{f}}=2\;log_{10}\left(\frac{D_{H}}{2\varepsilon }\right)+1.74$$

However, since a tunnel length with two different sections was considered for the present study, the 1D equation for both turbulent transport and molecular diffusion was solved numerically using MATLAB. A one-dimensional finite volume method has been employed for that purpose, using a third-order QUICK scheme for the convective terms. Preliminary tests showed that the explicit approach was sufficiently accurate for the temporal term. A typical mesh size of Δ*x* = 0.1 m was adopted for the longitudinal coordinate, with a time step of Δ*t* = 0.0025 s to assure stability and convergence. The effective diffusion coefficient was estimated using Equation (5), based on the friction factor computed in Equation (6).

For the 1D approach, the area of the ventilation pipeline was subtracted from the practical cross-sectional area of the tunnel, thus providing a slightly higher value of the uniform flow velocity for the convection phenomena, according to Equation (4). In the case of the 3D numerical simulation, this effect was also preserved through the geometrical inclusion of the ventilation duct along the tunnel, so three-dimensional mechanisms and swirl flows were fully modeled and solved.

In the 1D approach, the uniform flow velocity *u* was calculated as the ratio of the ventilation flow rate and the cross-sectional area of the tunnel. Note that, in this particular problem, this value differed from the top heading region to the main portion of the excavating tunnel due to the benching method. 

The effective diffusion coefficient was calculated using Equations (5) and (6), where the hydraulic diameter was computed according to its typical definition for flow in ducts, i.e., four times the ratio between the cross-sectional area of the flow and its wetted perimeter.

### 2.5. Numerical Modelling

The CFD software Ansys Fluent 17.0 was applied to simulate the propagation and dilution of toxic gases in the tunnel after blasting. This code uses the finite volume method and solves the 3D unsteady Reynolds-averaged Navier–Stokes (URANS) equations. The concentration of blasting fumes depends on the quantity of explosives and changes with time. In addition, the propagation and dilution of gases depends on the ventilation system used and the airflow rate. Therefore, the transient species transport model with diffusion energy sources was employed. The gas was defined as a mixture of CO, NO, NO_2_ and air, considering atmospheric conditions (101,325 Pa at 288 K). The pressure-implicit with splitting of operators (PISO) algorithm was used to solve the pressure–velocity coupling for the iterative process. The realizable k-ε turbulence model was also selected in this study [41]. 

**Figure 4:**
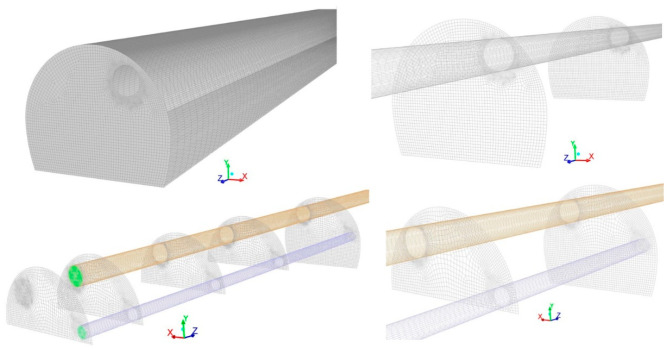
Model grid and boundary conditions, including the tunnel walls, cross-sectional areas and ventilation ducts (forced and exhaust systems).

To simulate different ventilation modes, two three-dimensional models of a 200 m-long railway tunnel with a cross-sectional area of 68 m^2^ were created and meshed using Ansys Gambit software. The model geometry and the meshing of the tunnel excavated using the top-heading-and-benching approach is shown in Figure 4. Different cross sections along the tunnel were selected to explore the CO, NO and NO_2_ concentration–time curves. In addition, three different ventilation systems were considered: forced, exhaust and mixed ventilation. Therefore, as shown in Figure 2, an air duct of 1800 mm in diameter was considered in the tunnel haunch in forced and exhaust models, and an additional short-forced duct of 1000 mm in diameter was included in the left wall in the mixed model. The initial concentration of the CO, NO and NO_2_ was defined as a boundary condition. The outlet of the air ducts close to the tunnel face (green area in Figure 4) was defined as the velocity inlet boundary condition and the tunnel exit was selected as the pressure outlet. In addition, the velocity inlet boundary condition was also selected at the inlet of the short-forced air duct in the mixed ventilation system. 

Finally, a wall roughness of 15 mm and a roughness constant of 0.8 were set. The entire geometry was meshed with 1,842,324 hexahedral elements in the forced and exhaust systems, and 1,748,215 elements in the mixed system. A finer mesh was defined in the air duct zone, and the grids have a higher density in these regions. The quality of the grid was measured using skewness and element quality indicators. A maximum skewness of 0.64 and an average element quality of 0.84 were obtained. To ensure the solution convergence, a fixed time step of 0.01 s and second-order discretization schemes were set. The residual values for convergence were fixed at 10^−5^ for all equations.

### 2.6. Grid Sensitivity Analysis

**Figure 5:**
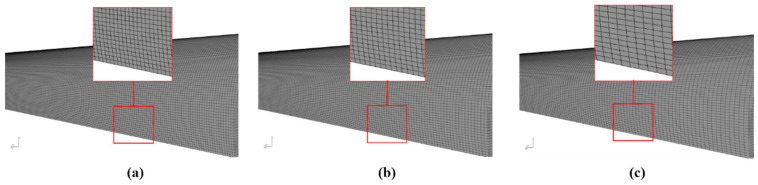
Grid sizes considered in the grid sensitivity analysis and details of the grid at the tunnel wall: (**a**) fine; (**b**) medium; and (**c**) coarse.

**Figure 6:**
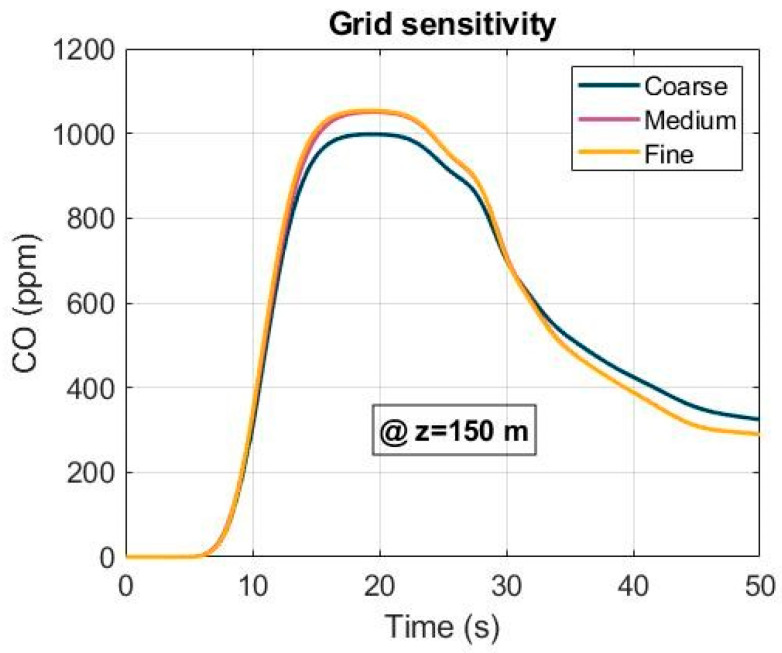
Grid sensitivity analysis of the CO concentration at Z = 150 m under forced ventilation.

A grid sensitivity analysis was conducted to improve the accuracy of the CFD simulations. Three mesh sizes—coarse (1,452,614 grids), medium (1,842,324 grids) and fine (2,235,452 grids)—were generated for a grid-independent study (Figure 5). The CO concentration was performed at the cross section of Z = 150 m under the forced ventilation mode and using an airflow of 40 m^3^ s^−1^. The results of the grid sensitivity study are presented in Figure 6 for the three mesh sizes. According to the results obtained, a medium-quality meshing was chosen to optimize the computing cost and efficiency.

### 2.7. Field Measurements and Model Validation

**Figure 7:**
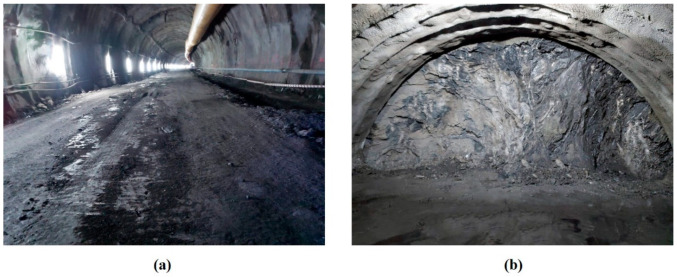
Railway tunnel excavated using the top-heading-and-benching method: (**a**) full face and forced duct, and (**b**) top heading.

**Figure 8:**
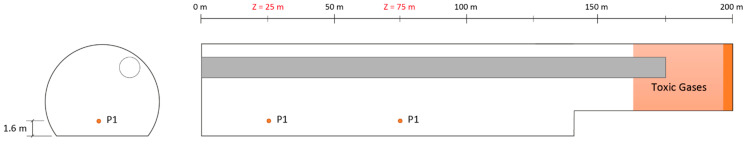
Location of the measuring points (P1) at the height of the human breathing zone (Y = 1.6 m) at the cross sections of Z = 25 and Z = 75 m.

To validate the accuracy of the CFD simulations, field measurements of the CO mass fraction were carried out in a railway tunnel excavated in a sandstone rock mass in the north of Spain (Figure 7). The tunnel portal was located at a height of 20 m above sea level with an air density of 1.2 kg m^−3^ and an air humidity of 72%. The tunnel was excavated using the top-heading-and-benching method, with a forced ventilation system and an airflow rate of 40 m^3^ s^−1^ in the working face. Steel arches, rock bolts and fiber-reinforced shotcrete were used for the support system. The length of the blast holes depends on the rock mass quality, reaching 2 m in this case study. Emulsion explosives were employed to excavate the top heading. An axial fan of type ZVN 1-16-250 was located at the tunnel portal with a power of 250 kW, equipped with frequency inverter, and an 1800 mm-diameter ventilation duct was used. The distance from the forced duct to the tunnel face was 25 m. The measurements were collected using a TROLEX TX 9165 stationary gas sensor with a range of 0–1000 ppm for CO in two cross sections, Z = 25 m and Z = 75 m, at a height of 1.6 m (Y = 1.6 m) in the tunnel axis (X = 0 m). The gas sensor had an accuracy of 1 ppm for CO and 0.1 ppm for NO_2_ with a response time (T_90_) < 15 s. Finally, the location of the measuring points is presented in Figure 8.

**Figure 9:**
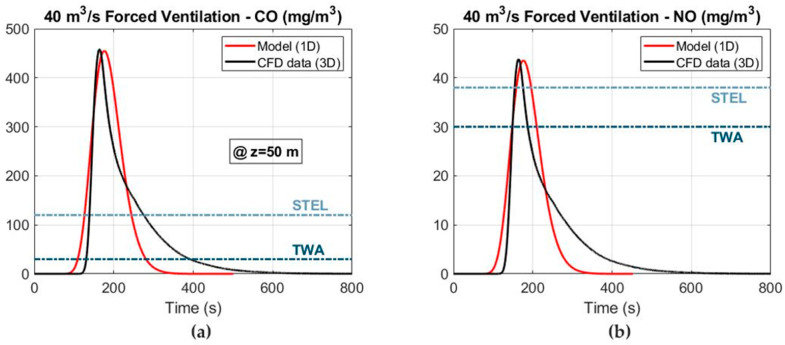
Comparative analysis between the 1D model and the 3D numerical simulations at the cross section of Z = 50 m under forced ventilation: (**a**) CO mass concentration and (**b**) NO mass concentration.

A comparative analysis between the CO and NO mass concentrations for the 1D mathematical model and the 3D numerical simulations are shown in Figure 9 at the cross section of Z = 50 m under forced ventilation and considering an airflow of 40 m^3^ s^−1^. In general, a good agreement is observed between the peak values of the CO and NO mass concentrations. The mass concentrations of CO (Figure 9a) and NO (Figure 9b) present Gaussian-like distributions with rapid growth and decline for the 1D model. However, rapid growth and slower decline are obtained in the 3D numerical simulations. 

**Figure 10:**
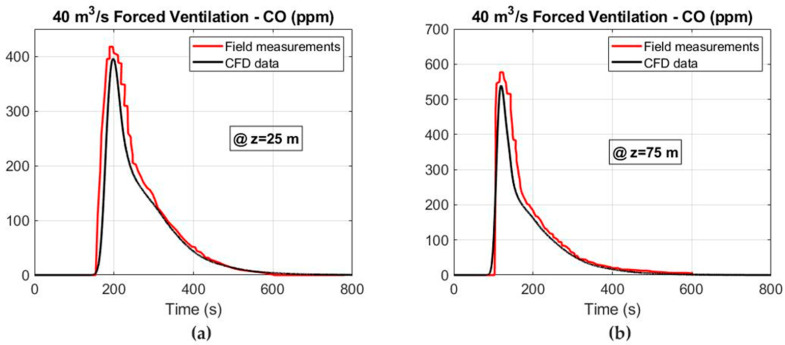
CFD results and field measurements considering an airflow of 40 m^3^ s^−1^ under the forced ventilation mode: (**a**) CO concentration at Z = 25 m and (**b**) CO concentration at Z = 75 m.

**Figure 11:**
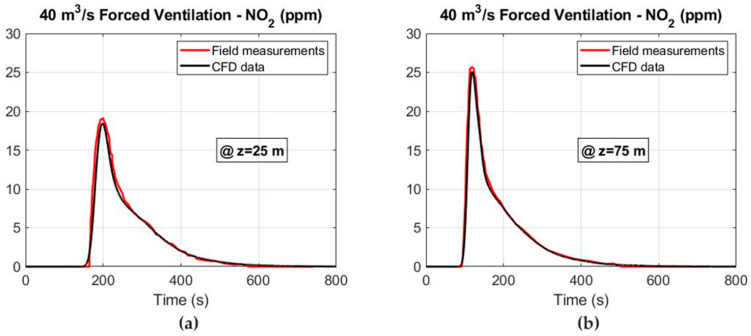
CFD results and field measurements considering an airflow of 40 m^3^ s^−1^ under the forced ventilation mode: (**a**) NO_2_ concentration at Z = 25 m and (**b**) NO_2_ concentration at Z = 75 m.

In addition, the CFD simulations and the field measurements are compared in Figure 10 and Figure 11 for the CO and NO_2_ concentrations at the cross sections of Z = 25 and Z = 75 m under forced ventilation. The results obtained show a good agreement between both the 3D numerical simulations and the measured values. Therefore, the numerical model that has been constructed is able to predict with good accuracy the migration of the noxious gases produced after blasting and to reliably determine the safe re-entry times.

## 3. Results and Discussion 

### 3.1. Toxic Gas Concentration

The volume of gas released per kilogram of explosive and the initial concentration of toxic gases are shown in Table 3. A blasting fume throwing length of 38.6 m was obtained by considering 2 m-long blast holes (medium quality rock mass) and an emulsion explosive mass of 118 kg. The volume of the tunnel occupied by the toxic gases immediately after blasting reached 1515 m^3^. Finally, a distance of 25 m between the mouth of the ventilation duct and the tunnel face was estimated.

### 3.2. 1D Model Results

**Figure 12:**
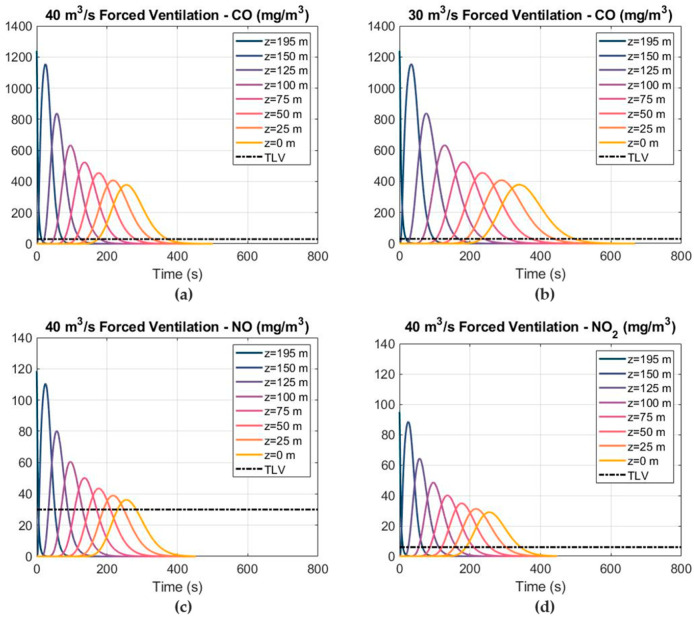
The results of the 1D model, showing the distribution of the toxic gas mass concentrations and TLVs at different cross sections and airflows under forced ventilation: (**a**) CO mass concentration at 40 m^3^ s^−1^; (**b**) CO mass concentration at 30 m^3^ s^−1^; (**c**) NO mass concentration at 40 m^3^ s^−1^; and (**d**) NO_2_ mass concentration at 40 m^3^ s^−1^.

The distribution of the mass concentrations for the toxic gases was obtained in the 1D mathematical model at different cross sections of the tunnel using forced ventilation, and is shown in Figure 12. A friction factor of 0.022 and effective diffusion coefficients of 1.9 and 1.45 m^2^ s^−1^ were estimated at the top heading and full face, respectively. The 25 ppm TLV (30 mg m^−3^ at working conditions) for CO has been shown to estimate the safe re-entry time according to safety and health regulations. In addition, TLVs of 25 and 3 ppm were also considered for NO and NO_2_, respectively. Due to the larger volume of CO released per kilogram of explosive, the CO concentration–time curve at the cross section of Z = 0 m was critical for predicting the re-entry time after blasting. Conversely, the results obtained show that the NO is the less critical gas, as it requires less ventilation time to be diluted after blasting. The CO, NO and NO_2_ concentration–time curves presented with a Gaussian-like distribution. After blasting, the supply fan was turned on and the peak values of CO, NO and NO_2_ moved towards the tunnel outlet and gradually decreased. The CO mass concentration–time curves are presented for airflows of 40 and 30 m^3^ s^−1^ in Figure 12a,b, respectively. The CO mass concentration was reduced from 1239 to 378 mg m^−3^ when the plug of blasting fumes reached the tunnel exit (Z = 0 m). The NO and NO_2_ mass concentrations also decreased when the blasting fumes were displaced towards the tunnel exit induced by the ventilation system.

### 3.3. Numerical Model Results

#### 3.3.1. Forced Ventilation

**Figure 13:**
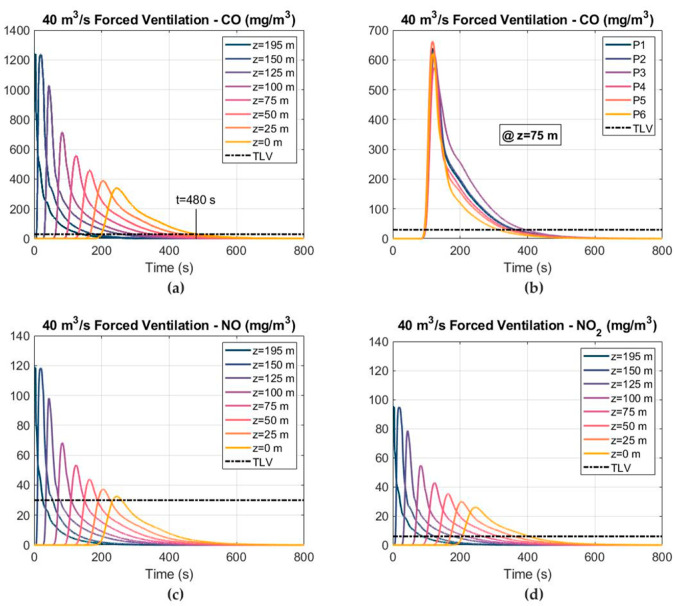
Distribution of the toxic gas mass concentrations and TLVs at different cross sections, considering an airflow of 40 m^3^ s^−1^ and forced ventilation: (**a**) CO mass concentration; (**b**) CO mass concentration at different points in the cross section of Z = 75 m; (**c**) NO mass concentration; and (**d**) NO_2_ mass concentration.

**Figure 14:**
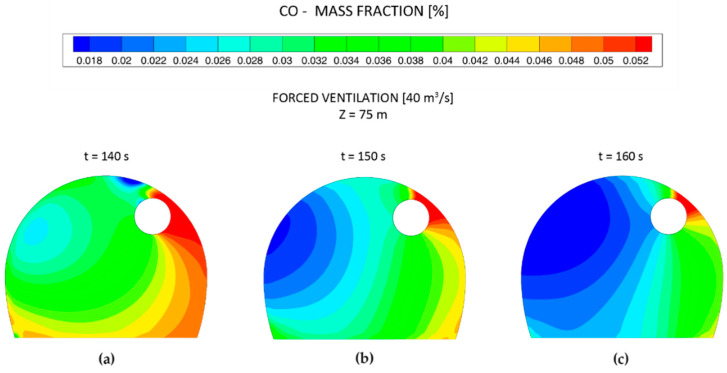
Distribution of the CO mass fraction at the cross section of Z = 75 m and different times, under forced ventilation and considering an airflow of 40 m^3^ s^−1^.

The distribution of the toxic gas mass concentrations at different cross sections, considering an airflow of 40 m^3^ s^−1^ and using the forced ventilation mode, is shown in Figure 13. As in the 1D model, the CO was the most critical gas and the re-entry time was determined based on the CO concentration–time curves. A ventilation time of 480 s was required after blasting to reduce the CO mass concentration below the TLV under the forced ventilation mode and considering an airflow of 40 m^3^ s^−1^. The CO mass concentration–time curves are shown in Figure 13b for the six observation points at the cross section of Z = 75 m. It can be observed that, due to the distribution of the backflow velocity in the cross section of the tunnel, the CO concentration increased at P3 and decreased at P6. The NO and NO_2_ mass concentrations are shown in Figure 13c,d, respectively. The distribution of the CO mass fraction at the cross section of Z = 75 m is shown in Figure 14 at different times after blasting. The CO mass fraction decreased with time and the peak values of the CO mass fraction were located at the right wall, where the air velocity was lower. Note that point P3 was located at the height of the workers’ breathing zone (Y = 1.6 m).

**Figure 15:**
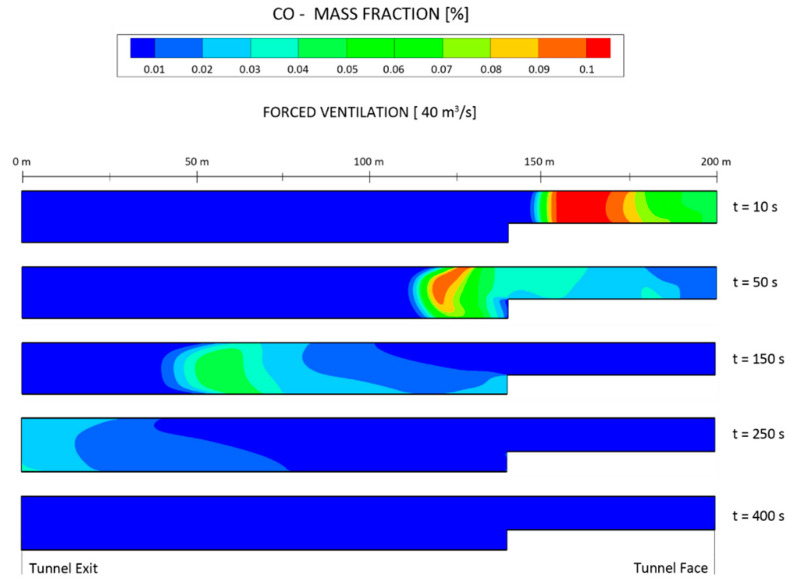
Distribution of the CO mass fraction at the longitudinal central section of X = 0 m at different times, under forced ventilation and considering an airflow of 40 m^3^ s^−1^.

Figure 15 shows the distribution of the CO mass fraction at the longitudinal central section of the tunnel at X = 0 m, from t = 10 s to t = 400 s, considering and airflow of 40 m^3^ s^−1^ and forced ventilation. The CO moved towards the tunnel exit and the mass fraction gradually decreased as the ventilation time increased. The CO was diluted from 0.1% to 0.06% when the ventilation time reached 150 s after blasting at the cross section of Z = 60 m. The air velocity at the outlet of the duct reached 15.72 m s^−1^ and gradually decreased as the air flowed towards the working face. After impacting the working face, some air continued towards the tunnel exit and some air returned again to the tunnel face induced by the jet, thus forming a vortex zone.

**Figure 16:**
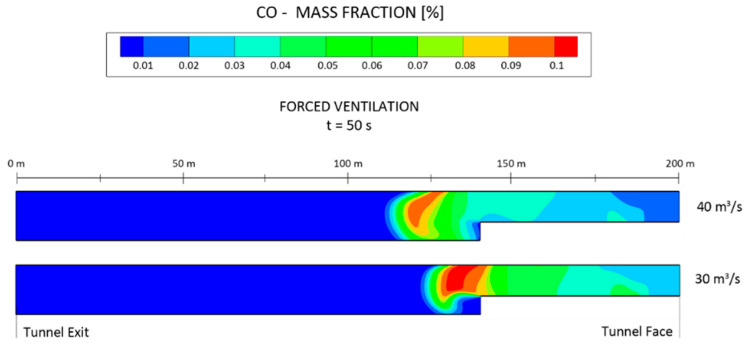
Comparative analysis of the distribution of the CO mass fraction at t = 50 s in the longitudinal central section (X = 0 m) under the forced ventilation mode and airflows of 30 and 40 m^3^ s^−1^.

A comparative analysis of the CO mass fraction between the airflows of 40 and 30 m^3^ s^−1^ using the forced ventilation system is presented in Figure 16. The CO mass fraction is shown at the longitudinal profile of the tunnel at t = 50 s. The toxic gases moved faster towards the tunnel outlet and the dispersion capacity increased as the airflow increased. The safe re-entry time was reduced by 25% when the airflow increased from 30 to 40 m^3^ s^−1^. 

**Figure 17:**
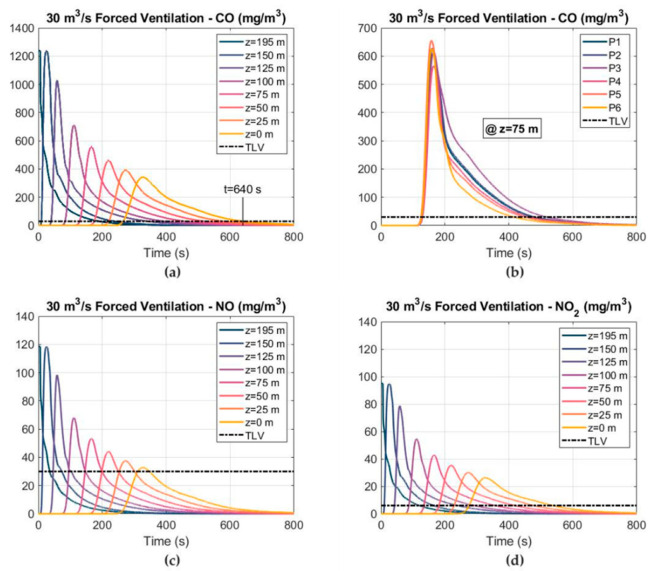
Distribution of the toxic gas mass concentrations and TLVs at different cross sections, considering an airflow of 30 m^3^ s^−1^ and the forced ventilation mode: (**a**) CO mass concentration; (**b**) CO mass concentration at different points in the cross section of Z = 75 m; (**c**) NO mass concentration; and (**d**) NO_2_ mass concentration.

The CO, NO and NO_2_ concentration–time curves, considering an airflow of 30 m^3^ s^−1^ and the forced ventilation mode, are depicted in Figure 17. The 25 ppm TLV level of CO and NO and the 3 ppm TLV level of NO_2_ are also shown. The ventilation time required to reduce the CO concentration below 25 ppm increased to 640 s when the airflow decreased to 30 m^3^ s^−1^. The peak values of the CO, NO and NO_2_ mass concentrations reached 342, 32 and 26 mg m^−3^ at the cross section of Z = 0. As can be observed, the decline stage after reaching the peak was longer when the airflow decreased. The CO mass concentration is shown in Figure 17b at the six observation points in the cross section of Z = 75 m. 

#### 3.3.2. Exhaust Ventilation

**Figure 18:**
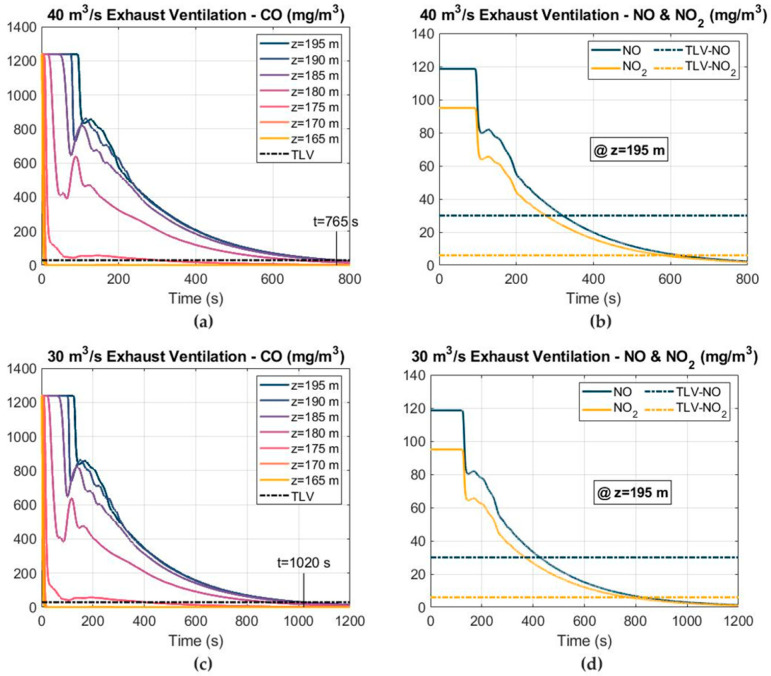
Distribution of the toxic gas mass concentrations and TLVs at different cross sections, under exhaust ventilation and using airflows of 30 and 40 m^3^ s^−1^: (**a**) CO mass concentration at 40 m^3^ s^−1^; (**b**) NO and NO_2_ mass concentrations at 40 m^3^ s^−1^; (**c**) CO mass concentration at 30 m^3^ s^−1^; and (**d**) NO and NO_2_ mass concentrations at 30 m^3^ s^−1^.

The CO, NO and NO_2_ concentration–time curves using the exhaust ventilation mode are shown in Figure 18, considering airflows of 30 and 40 m^3^ s^−1^ and an 1800 mm-diameter exhaust duct located at a distance of 25 m from the working face. Induced by the ventilation system, the toxic gases after blasting moved towards the tunnel exit within the ventilation duct and the fresh air entered through the tunnel itself towards the working face. In general, the results showed that the ventilation time required to reduce the concentration of the harmful gases increased when this ventilation mode was used compared to the typical forced ventilation. The safe re-entry time reached approximately 765 s using an airflow of 40 m^3^ s^−1^, increasing up to 1020 s if the airflow decreased to 30 m^3^ s^−1^.

#### 3.3.3. Mixed Ventilation

**Figure 19:**
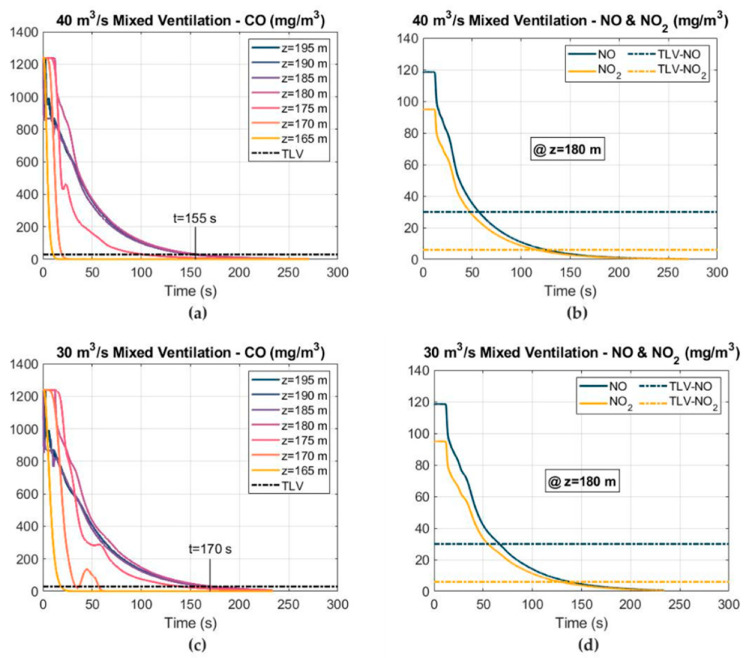
Distribution of the toxic gas mass concentrations and TLVs at different cross sections, under mixed ventilation and using airflows of 30 and 40 m^3^ s^−1^: (**a**) CO mass concentration at 40 m^3^ s^−1^; (**b**) NO and NO_2_ mass concentrations at 40 m^3^ s^−1^; (**c**) CO mass concentration at 30 m^3^ s^−1^; and (**d**) NO and NO_2_ mass concentrations at 30 m^3^ s^−1^.

A combination of a 1000 mm-diameter short-forced duct and an 1800 mm-diameter long-exhaust duct were considered in the mixed ventilation system. The simulations were conducted using airflows of 40 and 30 m^3^ s^−1^ for the exhaust duct and 15 m^3^ s^−1^ for the short-forced duct. In addition, the distance between the mouth of the short-forced duct and the tunnel face was 20 m (Z = 180 m), increasing up to 25 m (Z = 175 m) for the exhaust duct. The CO, NO and NO_2_ mass concentration–time curves are represented in Figure 19. As in the forced and exhaust ventilation modes, the CO was the most critical gas, requiring more ventilation time to reduce its concentration according to the safety and health regulations.

**Figure 20:**
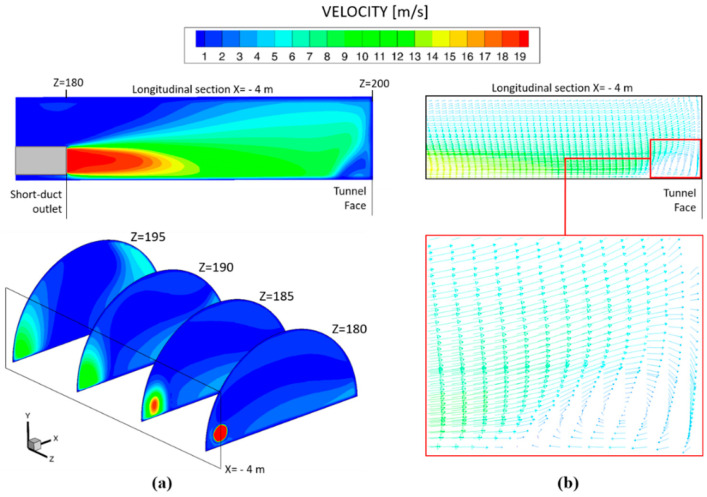
Mixed ventilation system: (**a**) blower jet at the outlet of the short-forced duct at the longitudinal section (X = − 4 m) and cross sections of Z = 180, Z = 185, Z = 190 and Z = 195 m, and (**b**) velocity vectors and details of the vortex zone at the longitudinal section of X = −4 m after impacting the tunnel face.

The air velocity at the outlet of the short-forced duct reached 19.10 m s^−1^, creating a vortex zone after impacting the working face and allowing toxic gases to be conducted to the tunnel exit more efficiently through the main exhaust duct (Figure 20). Based on the simulations, the mixed ventilation mode was the most effective system for reducing the concentrations of the toxic gases after blasting. 

The time required to reduce the CO concentration below the TLV strongly decreased compared to the other ventilation modes that were analyzed. The safe re-entry time reached 155 s using an exhaust duct airflow of 40 m^3^ s^−1^, and increased to 170 s when the airflow decreased to 30 m^3^ s^−1^. Therefore, the re-entry time can be reduced by 67% compared to the typical forced ventilation mode. The reduction in the required ventilation time by using the mixed ventilation mode represents a great improvement to the tunnel excavation cycle. This improvement could lead to a reduction in operating costs. However, the mixed ventilation mode has some drawbacks compared to the typical forced ventilation system. The main drawback is represented by the need to install a wire-reinforced exhaust duct and a dust collector, representing a significant increase in the operating and investment costs. Furthermore, an additional supply fan and a short-forced duct would be also required.

**Figure 21:**
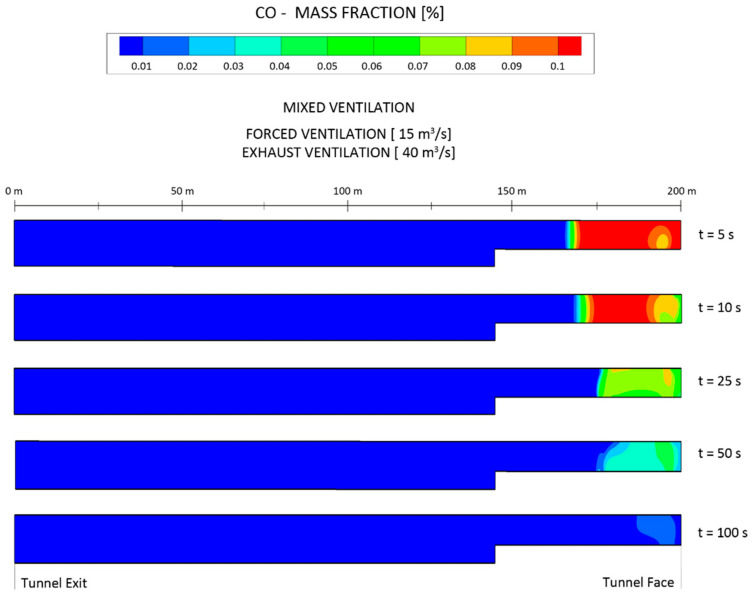
Distribution of the CO mass fraction at the longitudinal central section of X = 0 m at different times after blasting, under the mixed ventilation mode and using airflows of 15 and 40 m^3^ s^−1^ for the forced and exhaust ducts, respectively.

The distribution of the CO mass fraction at the longitudinal central section (X = 0 m) is shown in Figure 21 using the mixed ventilation mode and considering an airflow in the exhaust duct of 40 m^3^ s^−1^. The CO mass fraction reached 0.1% immediately after blasting, being continuously diluted with the increase in ventilation time. It can be seen that the mass fraction was reduced to 0.05% at t = 50 s. The jet effect at the outlet of the auxiliary short-forced duct can be observed mainly at t = 5 and t = 10 s, when the vortex zone was formed at the cross section of Z = 195 m. 

A summary of the results obtained for the re-entry time is presented in Table 4 for the three ventilation modes and airflows of 30 and 40 m^3^ s^−1^. As indicated previously, a significant reduction in the ventilation time can be obtained by using the mixed ventilation mode after blasting.

## 4. Conclusions

Toxic gases such as carbon monoxide and nitrogen oxides are released immediately after blasting by the detonation of explosives during the excavation of underground infrastructures. To provide a safe working environment, the concentrations of noxious gases must be reduced below the TLV according to health and safety regulations. One-dimensional mathematical models and three-dimensional CFD numerical simulations were conducted to analyze the migration of CO, NO and NO_2_ under different operating conditions. Forced, exhaust and mixed ventilation modes were compared to determine the safe re-entry times after blasting in a 200 m-long railway tunnel. Finally, to verify the accuracy of the CFD results, field measurements were carried out in a railway tunnel using gas sensors.

Based on the numerical simulations, CO was the most critical gas, as it required a longer ventilation time to reduce its concentration below the TLV. Therefore, the safe re-entry time was determined based on CO concentration–time curves. The safe re-entry time reached 480 s for the typical forced mode, and was reduced down to 155 s when mixed ventilation was used after blasting. The reduction in required ventilation time by using the mixed ventilation mode represents a great improvement in the tunnel excavation cycle. This improvement could lead to a reduction in operating costs. However, the mixed ventilation mode has some drawbacks compared to the typical forced system. The main drawback is the need to install a wire-reinforced exhaust duct and a dust collector, representing an increase in operating and investment costs.

The results obtained show that 1D models can be used to preliminary analyze the propagation of toxic gases. However, to reliably determine the safe re-entry time after blasting, 3D numerical models must be developed. A good agreement between both 3D numerical simulations and measured values was observed. Therefore, the numerical model that has been constructed is able to predict with a good accuracy the migration of the noxious gases released after blasting.

CFD numerical models can be also used to investigate the propagation and dilution of toxic gases in underground mines after blasting operations. The ventilation mode and the airflow rate used in the mining drifts must be considered. In addition, the cross-sectional area and the mass of explosives must be also analyzed. The ventilation system can be optimized to reduce the re-entry times, reducing the exploitation costs and therefore improving the mining cycle.

## Figures and Tables

**Table 1:** Exposure limits for CO, NO and NO_2_ [8].

Gases	TWA (ppm)	STEL (ppm)
CO	25	100
NO	30	200
NO_2_	3	5

**Table 2:** Exposure limits for CO according to international health and safety regulations [2,8,12].

Guidelines	TWA (ppm)	STEL (ppm)
ASM-2	25	100
NOHSC	30	200
NIOSH	35	200
OSHA PEL	35	200

**Table 3:** Initial concentrations of CO, NO and NO_2_.

Gases	Molar Mass (g mol ^−1^)	Volume of Gas (m^3^ kg^−1^)	Concentration (ppm)
CO	28.0	0.014	1053.74
NO	30.0	0.00125	100.80
NO_2_	46.1	0.00065	80.54

**Table 4:** Summary of the re-entry time results.

Ventilation Mode	Airflow (m^3^ s^−1^)	Re-Entry Time (s)
Forced	30	640
	40	480
Exhaust	30	1020
	40	765
Mixed	30	170
	40	155
